# Differences in characteristics of Medicare patients treated by ophthalmologists and optometrists

**DOI:** 10.1371/journal.pone.0227783

**Published:** 2020-09-14

**Authors:** Darby D. Miller, Michael W. Stewart, Joshua J. Gagne, Alan L. Wagner, Aaron Y. Lee

**Affiliations:** 1 Department of Ophthalmology, Mayo Clinic, Jacksonville, FL, United States of America; 2 Division of Pharmacoepidemiology and Pharmacoeconomics, Department of Medicine, Brigham and Women’s Hospital, Boston, MA, United States of America; 3 Department of Ophthalmology, Eastern Virginia Medical School, Norfolk, VA, United States of America; 4 Department of Ophthalmology, University of Washington, Seattle, WA, United States of America; University of Tennessee Health Science Center, UNITED STATES

## Abstract

**Purpose:**

To quantify differences in the age, gender, race, and clinical complexity of Medicare beneficiaries treated by ophthalmologists and optometrists in each of the United States.

**Design:**

Cross-sectional study based on publicly accessible Medicare payment and utilization data from 2012 through 2017.

**Methods:**

For each ophthalmic and optometric provider, demographic information of treated Medicare beneficiaries was obtained from the Medicare Provider Utilization and Payment Data from the Centers for Medicare and Medicaid Services (CMS) for the years 2012 through 2017. Clinical complexity was defined using CMS Hierarchical Condition Category (HCC) coding.

**Results:**

From 2012 through 2017, ophthalmologists in every state treated statistically significantly older beneficiaries, with the greatest difference (4.99 years in 2014) between provider groups seen in Rhode Island. In most states there was no gender difference among patients treated by the providers but in 46 states ophthalmologists saw a more racially diverse group of beneficiaries. HCC risk score analysis demonstrated that ophthalmologists in all 50 states saw more medically complex beneficiaries and the differences were statistically significant in 47 states throughout all six years.

**Conclusions:**

Although there are regional variations in the characteristics of patients treated by ophthalmologists and optometrists, ophthalmologists throughout the United States manage older, more racially diverse, and more medically complex Medicare beneficiaries.

## Introduction

Aging of the American population, with movement of the large post-world war II “baby boomers” cohort into the Medicare-covered age group, has substantially increased the prevalence of age-related eye diseases and the need for both primary and sub-specialty ophthalmology services [1, 2]. Advancements in imaging technology, ocular pharmacotherapy, and ophthalmic surgery have broadened the spectrum of treatable disease, further increasing the total amount of eye care delivered [2, 3]. Together, these factors suggest that a shortage of eye care providers may develop during the next 20 years. Several studies over the past 20 years have attempted to predict the future need for eye care providers, but their results have differed according to the relative proportions of provider groups used by each model [4].

Primary care and emergency department physicians treat some patients with acute ocular conditions, but most eye care in the United States is provided by ophthalmologists and optometrists in an outpatient setting. Diagnostic procedures and treatments are performed by both professions, but because of differences in education, training, and licensure, scopes of practice differ between the two specialties both within states and between states [5].

Not surprisingly, the geographic distribution of ophthalmologists and optometrists is not uniform within states, yet not that dissimilar. Some observers argue that regional access problems exist for the provision of some services and the performance of some procedures [5, 6]. Over the last several years, some states have expanded optometric scope of practice, ostensibly to improve patients’ access to eye care. Newly granted privileges include laser photocoagulation and photoablative procedures, intraocular injections, and eyelid surgeries [5]. Data showing that these expanded privileges have improved patient access to care is lacking and recent studies have concluded that access to care has not changed in states that have allowed optometrists to perform laser procedures (e.g. Oklahoma) [5–8]. Additionally, evidence showing that expanded privileges among optometrists increases their share of patients with more advanced eye diseases is lacking.

To better guide those state legislatures that are considering expansion of privileges for optometrists and other non-ophthalmologists, it is critical to understand the characteristics of patients who seek care from ophthalmologists and optometrists.

To our knowledge, no published study has described or evaluated the differences among Medicare beneficiaries treated by ophthalmologists and optometrists. The goal of this paper is to compare the ages, gender, race, and clinical complexity of Medicare beneficiaries seen by ophthalmologists and optometrists in each state from the years 2012 through 2017.

## Methods

### Background

This cross-sectional study evaluated the 2012 through 2017 Medicare Provider Utilization and Payment Data released by the Centers for Medicare and Medicaid Services (CMS) (https://data.cms.gov) (https://www.cms.gov/Research-Statistics-Data-and-Systems/Statistics-Trends-and-Reports/Medicare-Provider-Charge-Data/Physician-and-Other-Supplier2012.html). Institutional Review Board approval was not required since the data is publicly available online and all patient data had already been de-identified.

### Identification of ophthalmologists, optometrists, and patients

Ophthalmologists and optometrists were selected from the database and grouped together by provider specialty and by the National Provider Identifier (NPI). For each eye care provider and for each year in the database, the name, practice address, state, and total number of unique Medicare beneficiaries was extracted. The numbers of beneficiaries varied between states in accordance with the population size of each respective state. Information about the average age of the beneficiaries, the number of male and female beneficiaries, the number of Caucasian beneficiaries, and the average Risk Adjustment and Hierarchical Condition Category (HCC) coding score–used as a measure of clinical complexity—was collected.

The HCC score, which was developed and implemented by CMS in 2003, assigns a risk score to individuals based upon their chronic health conditions and demographic details [9, 10]. The score is ascertained through associated International Classification of Diseases (ICD) codes that are submitted by providers as part of the reimbursement process to the Medicare social health insurance system [9–13]. The HCC score is based on a complex risk adjustment model that predicts Medicare expenditures for the following year. The HCC diagnostic classification system has evolved over the years as diagnostic codes and groups have been updated but no major changes to the HCC score occurred during the study period.

Gender and racial percentages were calculated for each provider and year if the summed total was greater than 80% of the total reported unique Medicare beneficiaries for that provider and year. Otherwise, they were considered unreported. National- and state-level statistics were calculated for each demographic factor.

### Statistical analyses

Statistical analyses were performed using R (https://www.r-project.org/) software and Seaborn (https://seaborn.pydata.org/, Version 0.10.1). For each state and year, data associated with ophthalmologists and optometrists were grouped separately to compare the average age, gender, race, and HCC risk scores for the patient cohort seen by each type of provider. Standardized differences for ophthalmologists and optometrists were also calculated. For each of the figures, letter-value plots were used to visually display age, gender, race and HCC risk scores of ophthalmologists and optometrists [14]. Mann-Whitney U tests were performed to assess differences between groups.

## Results

### National data

**Fig 1:**
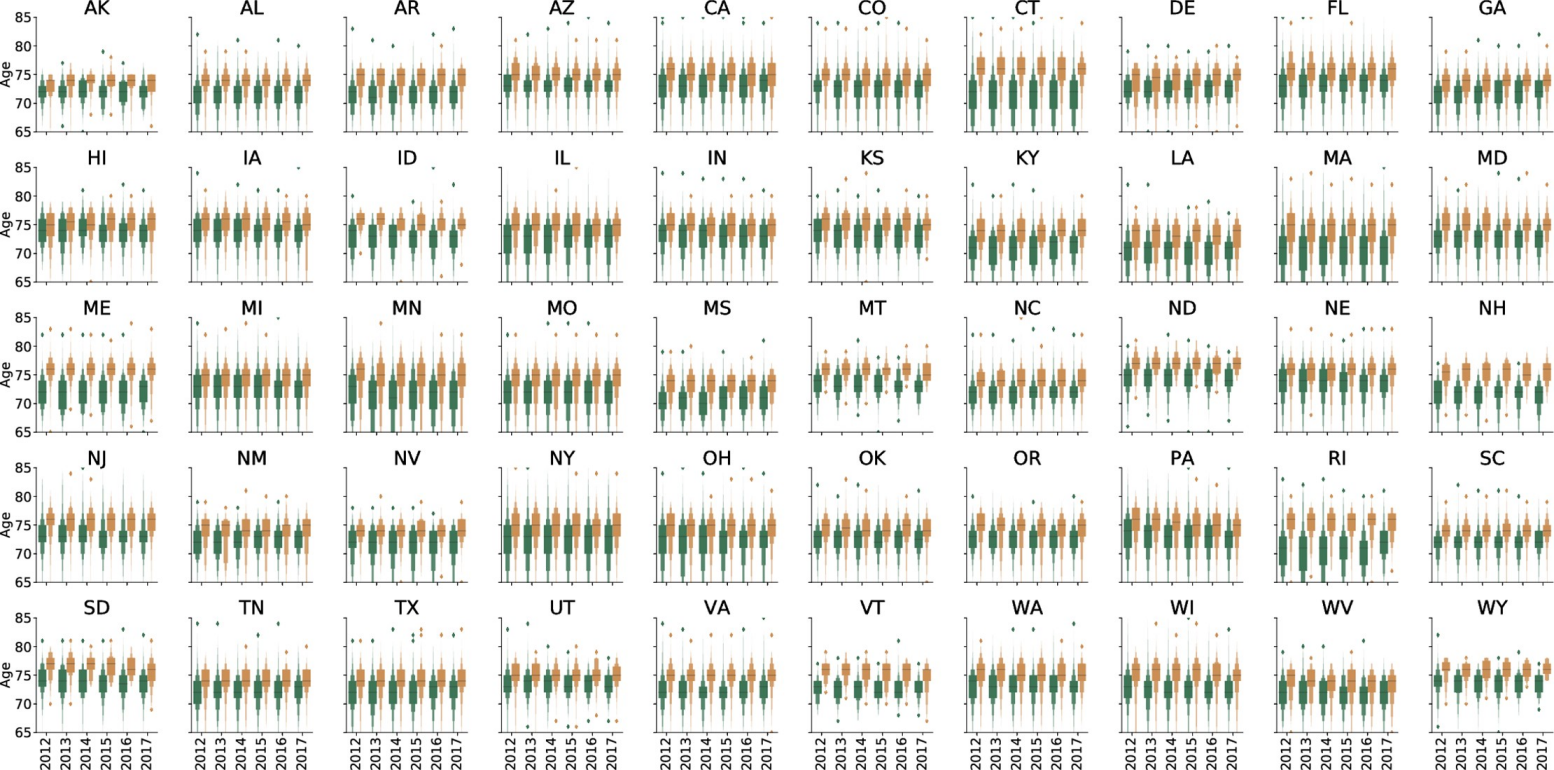
Letter-value plot for average age of Medicare beneficiary population by state and eye provider type. Ophthalmologists are shown in yellow and optometrists are shown in green. The black line represents the median value.

**Fig 2:**
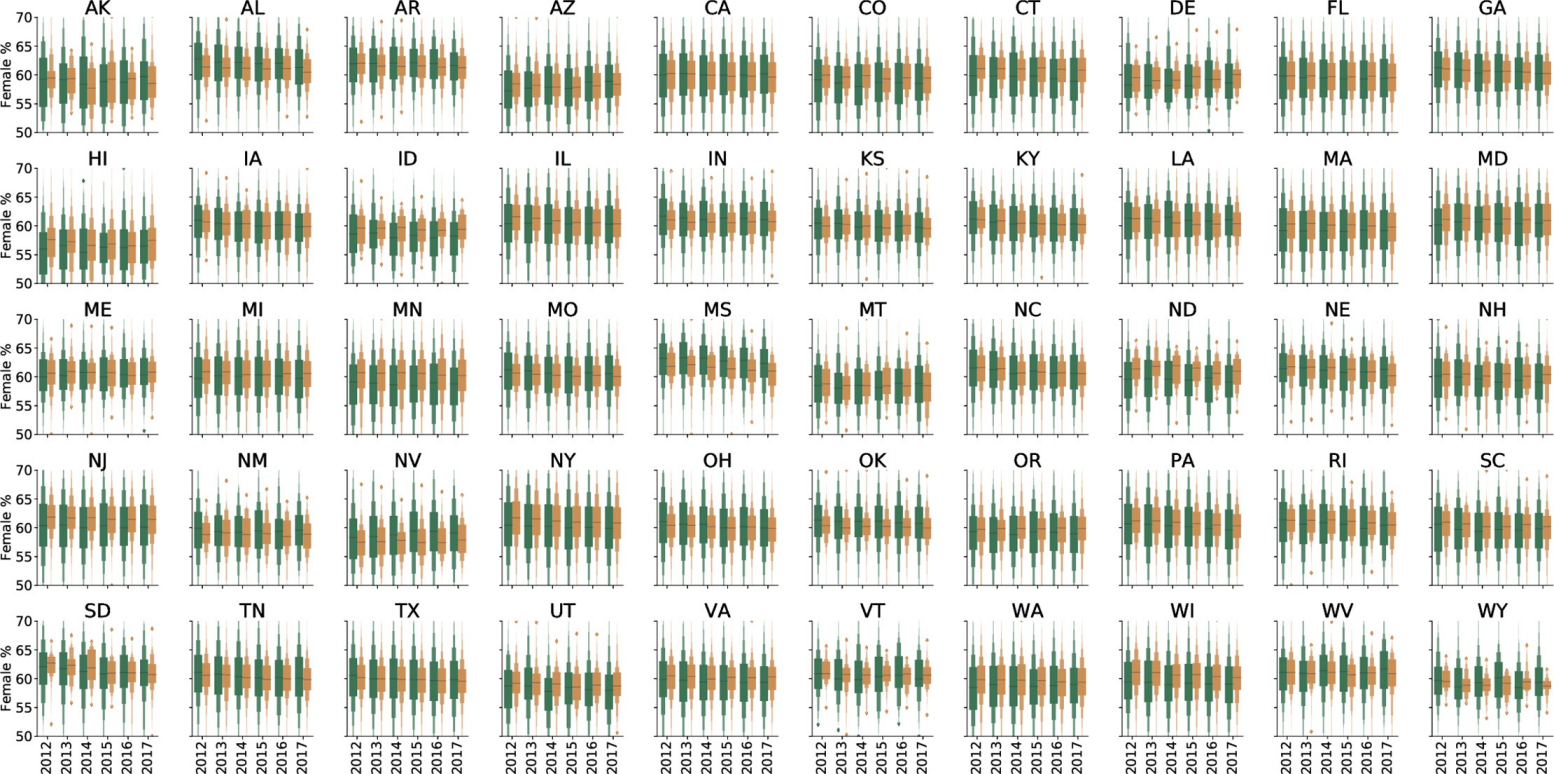
Letter-value plot for average percentage of female Medicare beneficiary population by state and eye provider type. Ophthalmologists are shown in yellow and optometrists are shown in green. The black line represents the median value.

**Fig 3:**
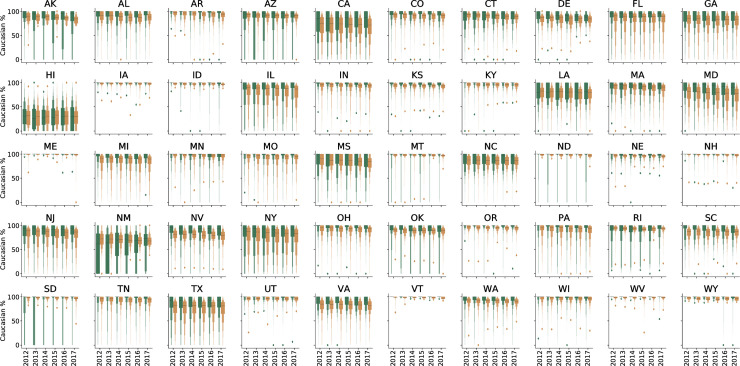
Letter-value plot for average percentage of Caucasian Medicare beneficiary population by state and eye provider type. Ophthalmologists are shown in yellow and optometrists are shown in green. The black line represents the median value.

**Fig 4:**
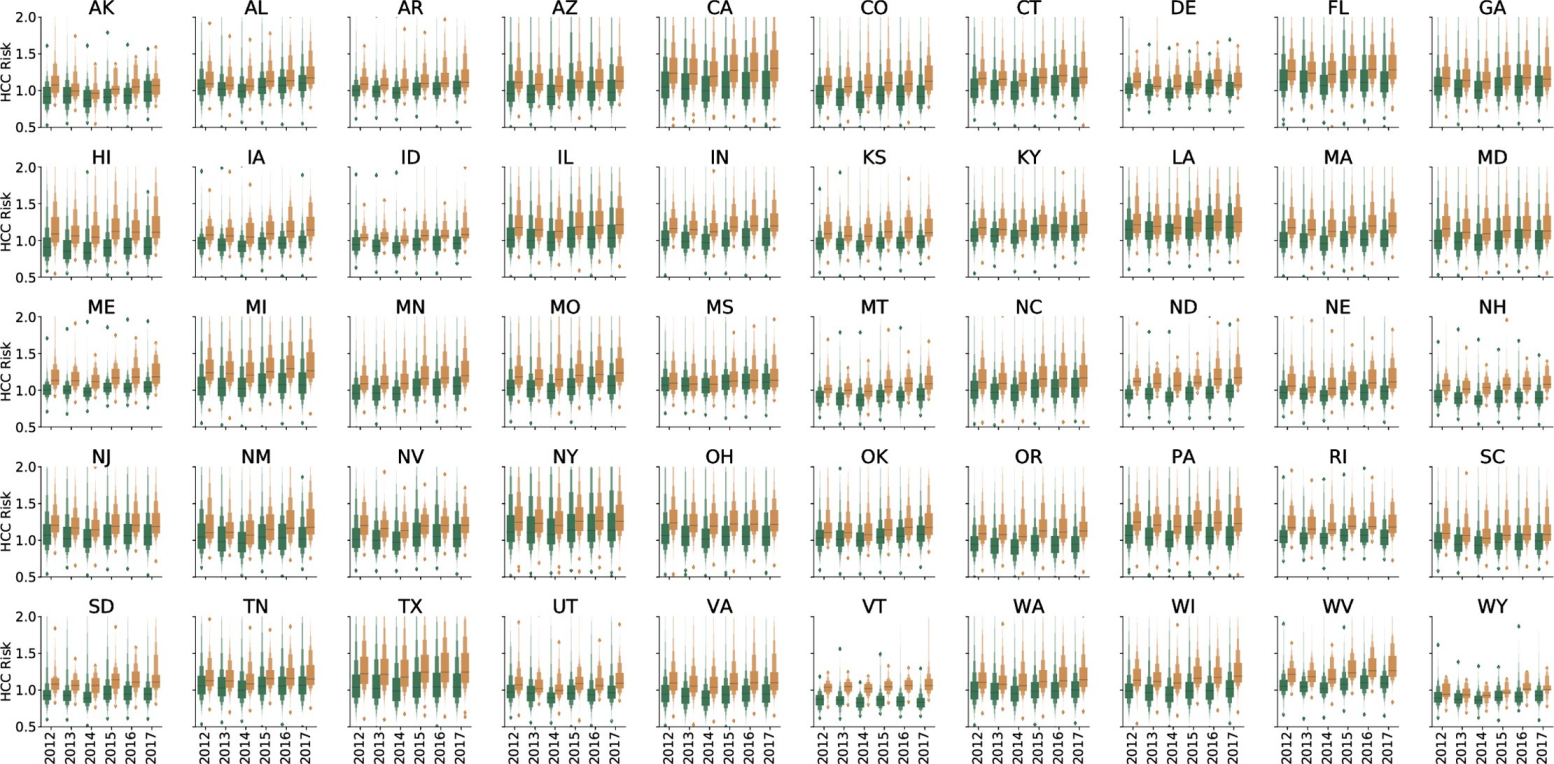
Letter value plot for average Hierarchical Condition Category (HCC) risk score by state and eye provider type. Ophthalmologists are shown in yellow and optometrists are shown in green. The black line represents the median value.

Data for patients seen by 20,487 unique ophthalmologists and 35,977 unique optometrists from 2012 through 2017 were included in the analysis (Table 1). Each ophthalmologist saw an average of 439.7 more Medicare beneficiaries each year compared to each optometrist. Nationally, beneficiaries cared for by ophthalmologists were older, more racially diverse, and had higher average HCC scores (Table 1 and Figs 1–4).

**Table 1:** Demographic characteristics of Medicare beneficiaries seen by optometrists (Optom) and ophthalmologists (Ophth) between 2012 and 2017 in the United States.

	**2012**			**2013**			**2014**			**2015**			**2016**			**2017**			**Total**		
	Optom.	Ophth.	SDD	Optom.	Ophth.	SDD	Optom.	Ophth.	SDD	Optom.	Ophth.	SDD	Optom.	Ophth.	SDD	Optom.	Ophth.	SDD	Optom.	Ophth.	SDD
**Providers (n)**	27123	17402	8	27900	17550		28469	17665		28834	17698		29227	17788		29790	17817		35977	20487	
**Number of Beneficiaries (Std Dev)**	197.8 (188.6)	457.2 (286.5)		198.5 (188.3)	456.8* (287.7)		223.7 (268.5)	733.8* (563.6)		227.0 (267.8)	732.0 (562.4)		232.1 (273.5)	734.1 (566.1)		234.3 (275.5)	722.3 (558.7)		219.5 (248.6)	659.2 (519.6)	
**HCC score (Std Dev)**	1.08 (0.29)	1.25 (0.28)	0.58***	1.06 (0.29)	1.22 (0.27)	0.58***	1.03 (0.29)	1.20 (0.27)	0.60***	1.09 (0.31)	1.26 (0.29)	0.60***	1.09 (0.32)	1.28 (0.30)	0.61***	1.09 (0.31)	1.29 (0.32)	0.62***	1.07 (0.30)	1.25 (0.29)	0.59***
**Age (Std Dev)**	72.4 (3.6)	74.7 (3.2)	0.67***	72.3 (3.6)	74.6 (3.1)	0.69***	72.2 (3.6)	74.6 (3.1)	0.70***	72.3 (3.4)	74.5 (3.1)	0.69***	72.3 (3.4)	74.5 (3.1)	0.69***	72.4 (3.2)	74.5 (3.0)	0.68***	72.3 (3.5)	74.6 (3.1)	0.69***
**Female % (Std Dev)**	60.1 (5.8)	60.6 (4.2)	0.10***	60.0 (5.7)	60.5 (4.1)	0.10***	59.8 (5.6)	60.2 (4.2)	0.08***	59.8 (5.6)	60.1 (4.1)	0.06***	59.8 (5.5)	60.1 (4.1)	0.06***	59.8 (5.4)	60.0 (4.2)	0.04***	59.87 (5.6)	60.23 (4.2)	0.07***
**Caucasian % (Std Dev)**	87.8 (23.5)	82.0 (22.9)	0.25***	87.7 (23.5)	81.6 (22.8)	0.26***	87.8 (23.3)	81.2 (22.5)	0.29***	88.2 (22.3)	80.8 (22.5)	0.33***	88.2 (21.9)	80.4 (22.3)	0.36***	88.4 (21.5)	80.1 (22.4)	0.38***	88.0 (22.6)	81.0 (22.6)	0.31***
**Black % (Std Dev)**	5.4 (13.5)	8.3 (14.5)	0.21 ***	5.4 (13.6)	8.4 (14.4)	0.21***	5.3 (13.5)	8.5 (14.2)	0.23***	5.3 (13.1)	8.6 14.4)	0.24***	5.4 (13.4)	8.6 (14.2)	0.23***	5.2 (12.8)	8.5 (14.0)	0.24***	5.3 (13.3)	8.5 (14.3)	0.23***
**Hispanic % (Std Dev)**	4.1 (14.0)	6.2 (14.4)	0.15***	4.1 (13.8)	6.3 (14.1)	0.15***	3.9 (13.1)	6.3 (14.1)	0.18***	3.8 (12.6)	6.3 (14.0)	0.19***	3.6 (12.0)	6.3 (13.6)	0.21***	3.5 (11.5)	6.3 (13.8)	0.23***	3.8 (12.8)	6.3 (14.0)	0.19***
**Asian PI % (Std Dev)**	1.6 (9.6)	2.9 (10.5)	0.13***	1.5 (9.3)	3.0 (10.6)	0.15***	1.6 (9.4)	3.1 (10.5)	0.15***	1.4 (8.5)	3.2 (10.6)	0.18***	1.3 (8.1)	3.3 (10.6)	0.21***	1.4 (8.3)	3.4 (10.8)	0.21***	1.5 (8.9)	3.2 (10.6)	0.17***
**Nat Am % (Std Dev)**	1.0 (9.6)	0.2 (2.3)	0.13***	1.1 (10.0)	0.2 (2.2)	0.13***	1.2 (10.5)	0.2 (2.6)	0.14***	1.1 (9.6)	0.2 (2.4)	0.13***	1.1 (9.6)	0.2 (2.4)	0.12***	1.1 (9.5)	0.2 (1.9)	0.13***	1.1 (9.8)	0.2 (2.3)	0.13***

Listed in the table are: number of providers, mean number of beneficiaries, HCC score, age in years, female percentage, and race (Caucasian, Black, Hispanic, Asian/Pacific Islander (Asian PI), and Native American (Nat Am)). SDD = standardized difference. (Std Dev) = standard deviation.

Note

*** P < 0.01.

### State age, gender, racial, and HCC data

In all states during the study period, ophthalmologists saw a larger number of elderly patients compared to optometrists (Fig 1 and S1 Fig). The youngest patient population was seen by optometrists in the state of Louisiana in 2015 and the oldest patient population was seen by ophthalmologists in North Dakota in 2015. The largest difference in ages between men and women was in Rhode Island in 2014 (female–male = 4.99 years).

In 41 of the 50 states there was no statistically significant difference in the genders of patients seen by eye care providers from 2012 through 2017 (Fig 2 and S2 Fig). In Minnesota, New Jersey, New York, Wisconsin, Connecticut, and Pennsylvania, ophthalmologists saw a higher percentage of females than did optometrists during each of the six years. In Alabama, Oklahoma, and Mississippi, this trend was reversed, as optometrists saw more females than did ophthalmologists.

In 42 states during each of the six years there was a statistically significant difference in the racial composition of the beneficiaries seen by ophthalmologists and optometrists, with the exception of Native Americans (Table 1 and Fig 3 and S3 Fig). In every state except for Arizona, Montana, North Dakota, and South Dakota, ophthalmologists saw more non-Caucasian patients than did optometrists. The states without a statistically significant difference were Hawaii, New Mexico, Louisiana, Mississippi, Alaska, Delaware, Rhode Island, and Connecticut.

During every year in every state ophthalmologists treated more medically complex patients compared to optometrists (Fig 4). This difference was statistically significant in every state except for Mississippi, Wyoming, and Alaska (S4 Fig). The greatest difference was in Vermont in 2017. Scope of practice laws changed in Louisiana in 2014, allowing optometrists to perform certain types of eye and eyelid surgery. When HCC scores for optometrists before and after 2014 were compared, there was no statistically significant difference (*P* = 0.22). There was, however, a statistically significant increase in HCC scores for ophthalmologists in Louisiana when comparing after 2014 to before 2014 (*P* < 0.05), indicating an increase in clinical complexity of patients being seen.

## Discussion

To the best of our knowledge this is the first study to compare patients seen by ophthalmologists and optometrists according to their ages, gender, race, and clinical complexity. According to Medicare payment and utilization data from 2012 through 2017, ophthalmologists saw older, sicker, and more racially diverse patients than optometrists.

Hierarchical Condition Category Coding was introduced by CMS as a risk-adjustment approach to estimate future health care costs for patients [6] HCC coding relies on ICD coding to assign risk scores to patients. The ICD codes and additional risk factors for future health care costs, such as gender, age, living situation, and Medicaid eligibility, are used to calculate a Risk Adjustment Factor (RAF) that is assigned to each HCC category. Overall, HCC coding summarizes each patient’s complexity by way of a single numerical value. A patient with few serious health care conditions would have a low RAF but a patient with several chronic conditions would be expected to use more health care services and incur higher health care costs. Patients who are healthier than average will have HCC scores less than 1.00, whereas those that are less healthy than average have HCC scores greater than 1.00. As the healthcare landscape has increasingly shifted to value-based payment models, HCC coding has become more prevalent. Insurance companies often use the RAF to predict future health care costs.

The higher proportion of high-complexity patients seeking specialist care from ophthalmologists can be explained, at least in part, by several factors. The incidences of the most common vision threatening conditions in the United States (cataracts, age-related macular degeneration, glaucoma, and diabetic retinopathy) increase with patient age; therefore, it would be expected that elderly patients have higher HCC risk scores. It may be that many elderly patients recognize that ophthalmologists, with their medical school education, and medical and surgical residency training, are better equipped than optometrists to prescribe pharmacotherapies and perform surgeries for complex conditions [15]. Many patients with age-related vision threatening diseases may have initially presented to optometrists or general physicians but required referral to ophthalmologists. Funneling of complex patients to ophthalmologists would be expected to increase the HCC scores among ophthalmologic practices.

Optometric practices are often affiliated with retail optical chains. These may attract younger, more mobile patients than ophthalmology practices, many of which are located in multi-specialty medical centers or on hospital campuses. Due to their respective levels of education and training, optometry practices are known for their expertise in contact lenses and glasses whereas ophthalmologists are known for treating complex medical and surgical conditions.

This study shows that ophthalmologists manage a more racially diverse group of Medicare beneficiaries than do optometrists. One might postulate that more ophthalmologists practice in urban areas, which are more racially diverse than rural areas, but the percentages of ophthalmologists and optometrists that practice in urban and rural locations are similar [6].

Several limitations of this study need to be mentioned. While the strength of the study lies in the large numbers of practitioners and patients identified from the large cohort of United States Medicare beneficiaries, it has the inherent weaknesses of a retrospective study, including data that was collected after the encounters. As with any database analysis, the validity of the results depends on the accuracy of the underlying data. Data for all variables were not fully reported for each state in each year. Moreover, ICD codes from Medicare claims data may not capture with perfect sensitivity all the comorbid conditions that patients have. We examined patient age, gender, race, and HCC risk score averages by state, but not at county, city, or individual practitioner levels.

An additional limitation of this study is that HCC scores, by design, are generalized measures of patient morbidity and not specific to eye conditions. The HCC score includes both eye-related diseases, such as macular degeneration, and non-eye-related diseases, such as hip fractures. Nevertheless, certain non-eye-related diseases must be taken into account when performing eye exams because they have ocular manifestations and can impact treatment plans. Several ocular diseases, such as giant cell arteritis and scleritis, require treatment with systemic glucocorticoids that have numerous ocular and systemic side effects [16, 17]. For example, a serious adverse event caused by long-term corticosteroid use is osteonecrosis, which can lead to fractures [18, 19]. Thus, the understanding of medically complex diseases and pharmacology, which is part of a general medical education and residency training, provides ophthalmologists with the knowledge and experience to evaluate and treat the whole patient.

The data from this study show that ophthalmologists and optometrists see patient populations that differ with respect to age, race, and disease severity. The national data show that ophthalmologists care for a larger percentage of elderly patients with more severe medical conditions. Health policy makers should understand that the two professions cannot be viewed interchangeably as some would suggest and that decisions to remove scope of practice barriers should be considered carefully. Importantly, in Louisiana, the one state where scope of practice was expanded during the time period of this study (2014), there was a statistically significant increase in HCC scores of patients seen by ophthalmologists (*P* < 0.05) but not optometrists (*P* = 0.22). Thus, when scope of practice is expanded for optometrists, it does not change the complexity of patients they see. Future studies that compare outcomes of care provided by ophthalmologists to those of optometrists should consider the differences in patient characteristics that we observed.

## Supporting information

S1 Fig Statistical significance test for average age comparing differences between ophthalmologists and optometrists for each state and year. The red dashed line represents the threshold for statistical significance. The lowest possible value is 2.2 x 10^−16^ and most of the P values are too small to be seen on this scale.
(DOCX)

S2 Fig Statistical significance for percent of female beneficiaries comparing differences between ophthalmologists and optometrists for each state and year. The red dashed line represents the threshold for statistical significance. The lowest possible value is 2.2 x 10^−16^.
(DOCX)

S3 Fig Statistical significance for percent of Caucasian beneficiaries comparing differences between ophthalmologists and optometrists for each state and year. The red dashed line represents the threshold for statistical significance. The lowest possible value is 2.2 x 10^−16^ and most of the P values are too small to be seen on this scale.
(DOCX)

S4 Fig Statistical significance for Hierarchical Condition Category risk score comparing differences between ophthalmologists and optometrists for each state and year. The red dashed line represents the threshold for statistical significance. The lowest possible value is 2.2 x 10^−16^ and most of the P values are too small to be seen on this scale.
(DOCX)

S1 File (DOCX)
