# Supplementary material for: Differences in characteristics of Medicare patients treated by ophthalmologists and optometrists
Source: PLoS One. 2020 Sep 14;15(9):e0227783. doi: 10.1371/journal.pone.0227783 (PMC7489526; doi:10.1371/journal.pone.0227783)
Supplement: S2 Fig — The red dashed line represents the threshold for statistical significance. The lowest possible value is 2.2 x 10^−16^. (DOCX) [file pone.0227783.s002.docx]

**
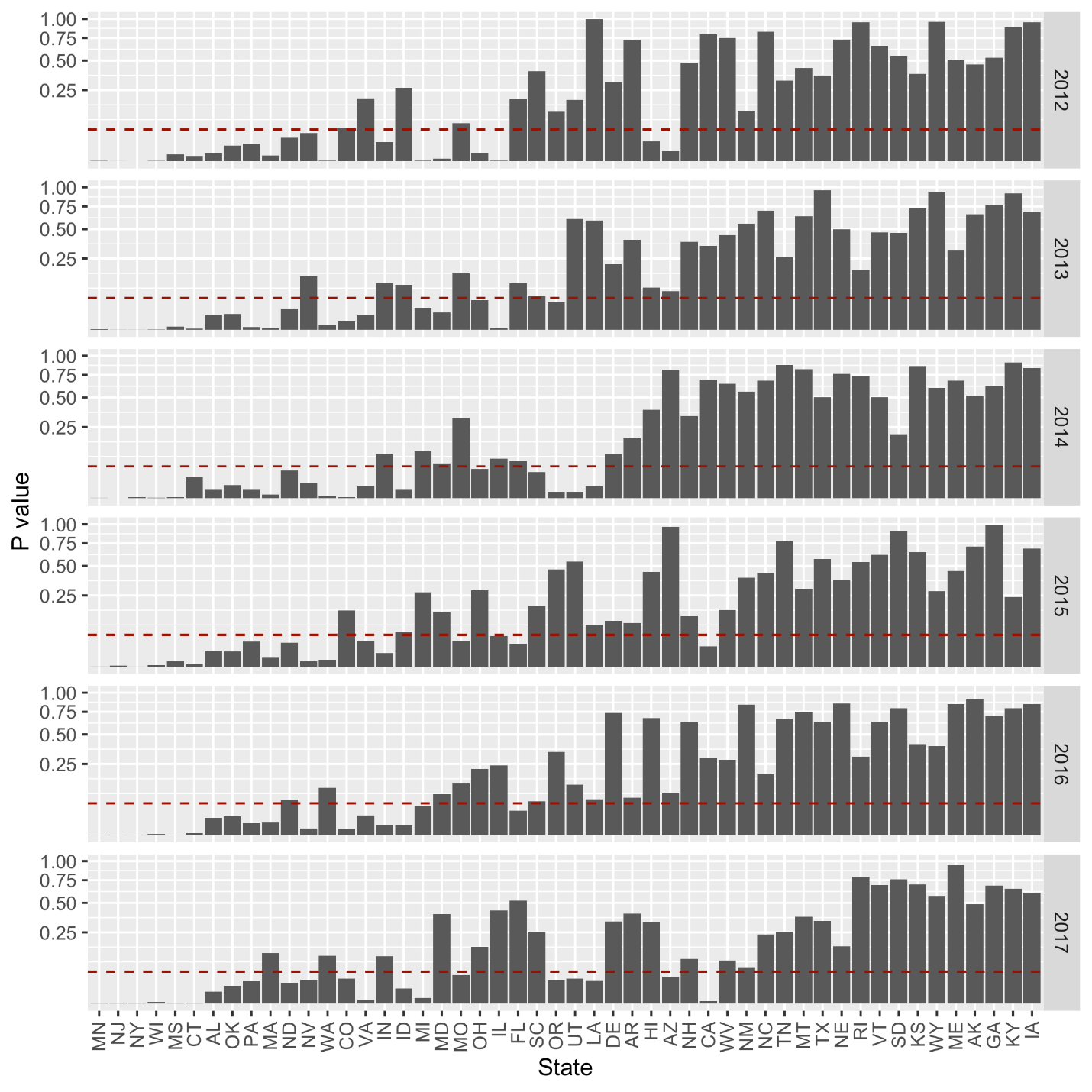
**

**S2 Fig: Statistical significance for percent of female beneficiaries comparing differences between ophthalmologists and optometrists for each state and year.** The red dashed line represents the threshold for statistical significance. The lowest possible value is 2.2 x 10^-16^.
