# Supplementary material for: Differences in characteristics of Medicare patients treated by ophthalmologists and optometrists
Source: PLoS One. 2020 Sep 14;15(9):e0227783. doi: 10.1371/journal.pone.0227783 (PMC7489526; doi:10.1371/journal.pone.0227783)
Supplement: S3 Fig — The red dashed line represents the threshold for statistical significance. The lowest possible value is 2.2 x 10^−16^ and most of the P values are too small to be seen on this scale. (DOCX) [file pone.0227783.s003.docx]

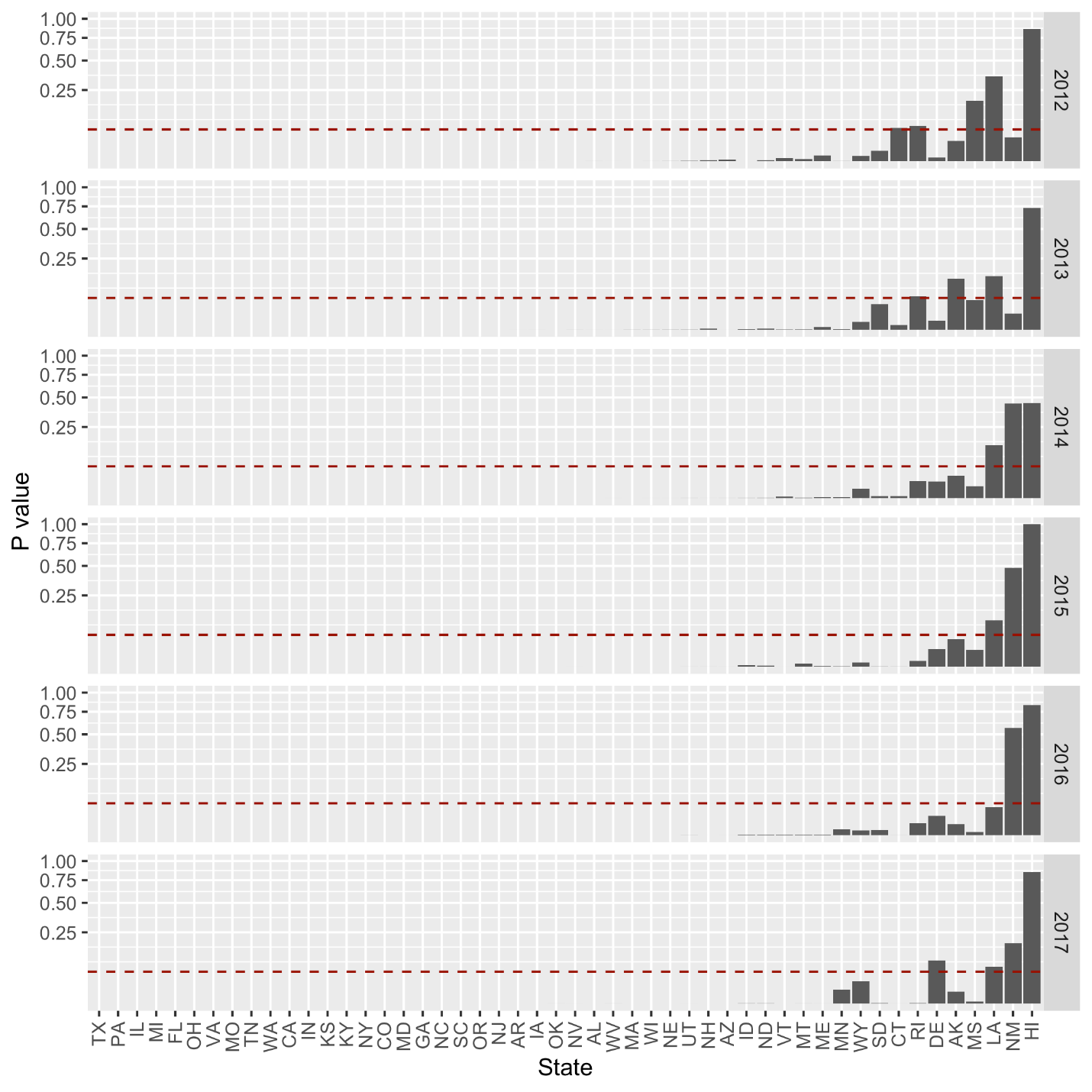


**S3 Fig: Statistical significance for percent of Caucasian beneficiaries comparing differences between ophthalmologists and optometrists for each state and year.** The red dashed line represents the threshold for statistical significance. The lowest possible value is 2.2 x 10^-16^ and most of the P values are too small to be seen on this scale.
