# Supplementary material for: Differences in characteristics of Medicare patients treated by ophthalmologists and optometrists
Source: PLoS One. 2020 Sep 14;15(9):e0227783. doi: 10.1371/journal.pone.0227783 (PMC7489526; doi:10.1371/journal.pone.0227783)
Supplement: S1 File — (DOCX) [file pone.0227783.s005.docx]

[1] "BENEFICIARY_AVERAGE_AGE" "2012"

provtype value

1 Ophthalmology 74.66165

2 Optometry 72.38137

provtype value

1 Ophthalmology 3.166108

2 Optometry 3.626541

Welch Two Sample t-test

data: sdat$value by sdat$provtype

t = 70.007, df = 40579, p-value < 2.2e-16

alternative hypothesis: true difference in means is not equal to 0

95 percent confidence interval:

2.216433 2.344116

sample estimates:

mean in group Ophthalmology mean in group Optometry

74.66165 72.38137

[1] 0.6698576

[1] "BENEFICIARY_AVERAGE_AGE" "2013"

provtype value

1 Ophthalmology 74.59117

2 Optometry 72.25455

provtype value

1 Ophthalmology 3.14462

2 Optometry 3.61109

Welch Two Sample t-test

data: sdat$value by sdat$provtype

t = 72.777, df = 40995, p-value < 2.2e-16

alternative hypothesis: true difference in means is not equal to 0

95 percent confidence interval:

2.273686 2.399546

sample estimates:

mean in group Ophthalmology mean in group Optometry

74.59117 72.25455

[1] 0.6901024

[1] "BENEFICIARY_AVERAGE_AGE" "2014"

provtype value

1 Ophthalmology 74.55737

2 Optometry 72.21599

provtype value

1 Ophthalmology 3.095715

2 Optometry 3.562579

Welch Two Sample t-test

data: sdat$value by sdat$provtype

t = 74.477, df = 41313, p-value < 2.2e-16

alternative hypothesis: true difference in means is not equal to 0

95 percent confidence interval:

2.279765 2.403003

sample estimates:

mean in group Ophthalmology mean in group Optometry

74.55737 72.21599

[1] 0.701576

[1] "BENEFICIARY_AVERAGE_AGE" "2015"

provtype value

1 Ophthalmology 74.52023

2 Optometry 72.26933

provtype value

1 Ophthalmology 3.086895

2 Optometry 3.428265

Welch Two Sample t-test

data: sdat$value by sdat$provtype

t = 73.182, df = 40417, p-value < 2.2e-16

alternative hypothesis: true difference in means is not equal to 0

95 percent confidence interval:

2.190608 2.311179

sample estimates:

mean in group Ophthalmology mean in group Optometry

74.52023 72.26933

[1] 0.6900245

[1] "BENEFICIARY_AVERAGE_AGE" "2016"

provtype value

1 Ophthalmology 74.50770

2 Optometry 72.29025

provtype value

1 Ophthalmology 3.057533

2 Optometry 3.382547

Welch Two Sample t-test

data: sdat$value by sdat$provtype

t = 73.226, df = 40484, p-value < 2.2e-16

alternative hypothesis: true difference in means is not equal to 0

95 percent confidence interval:

2.158102 2.276811

sample estimates:

mean in group Ophthalmology mean in group Optometry

74.50770 72.29025

[1] 0.6877672

[1] "BENEFICIARY_AVERAGE_AGE" "2017"

provtype value

1 Ophthalmology 74.50968

2 Optometry 72.40060

provtype value

1 Ophthalmology 3.017411

2 Optometry 3.205439

Welch Two Sample t-test

data: sdat$value by sdat$provtype

t = 72.09, df = 39280, p-value < 2.2e-16

alternative hypothesis: true difference in means is not equal to 0

95 percent confidence interval:

2.051735 2.166420

sample estimates:

mean in group Ophthalmology mean in group Optometry

74.50968 72.40060

[1] 0.6775402

[1] "ayl_female_perc" "2012"

provtype value

1 Ophthalmology 60.57378

2 Optometry 60.08873

provtype value

1 Ophthalmology 4.212556

2 Optometry 5.766400

Welch Two Sample t-test

data: sdat$value by sdat$provtype

t = 9.8565, df = 41047, p-value < 2.2e-16

alternative hypothesis: true difference in means is not equal to 0

95 percent confidence interval:

0.3885968 0.5815086

sample estimates:

mean in group Ophthalmology mean in group Optometry

60.57378 60.08873

[1] 0.09605757

[1] "ayl_female_perc" "2013"

provtype value

1 Ophthalmology 60.45309

2 Optometry 59.96047

provtype value

1 Ophthalmology 4.150381

2 Optometry 5.682807

Welch Two Sample t-test

data: sdat$value by sdat$provtype

t = 10.263, df = 41814, p-value < 2.2e-16

alternative hypothesis: true difference in means is not equal to 0

95 percent confidence interval:

0.3985477 0.5867074

sample estimates:

mean in group Ophthalmology mean in group Optometry

60.45309 59.96047

[1] 0.09900192

[1] "ayl_female_perc" "2014"

provtype value

1 Ophthalmology 60.20115

2 Optometry 59.82993

provtype value

1 Ophthalmology 4.195185

2 Optometry 5.601564

Welch Two Sample t-test

data: sdat$value by sdat$provtype

t = 7.8127, df = 42147, p-value = 5.727e-15

alternative hypothesis: true difference in means is not equal to 0

95 percent confidence interval:

0.2780896 0.4643500

sample estimates:

mean in group Ophthalmology mean in group Optometry

60.20115 59.82993

[1] 0.07501526

[1] "ayl_female_perc" "2015"

provtype value

1 Ophthalmology 60.13023

2 Optometry 59.81472

provtype value

1 Ophthalmology 4.144181

2 Optometry 5.561477

Welch Two Sample t-test

data: sdat$value by sdat$provtype

t = 6.7287, df = 42413, p-value = 1.733e-11

alternative hypothesis: true difference in means is not equal to 0

95 percent confidence interval:

0.2236003 0.4074068

sample estimates:

mean in group Ophthalmology mean in group Optometry

60.13023 59.81472

[1] 0.06433206

[1] "ayl_female_perc" "2016"

provtype value

1 Ophthalmology 60.06985

2 Optometry 59.79508

provtype value

1 Ophthalmology 4.120195

2 Optometry 5.502140

Welch Two Sample t-test

data: sdat$value by sdat$provtype

t = 5.9391, df = 42693, p-value = 2.889e-09

alternative hypothesis: true difference in means is not equal to 0

95 percent confidence interval:

0.1840917 0.3654533

sample estimates:

mean in group Ophthalmology mean in group Optometry

60.06985 59.79508

[1] 0.05653136

[1] "ayl_female_perc" "2017"

provtype value

1 Ophthalmology 59.95618

2 Optometry 59.76416

provtype value

1 Ophthalmology 4.214050

2 Optometry 5.432747

Welch Two Sample t-test

data: sdat$value by sdat$provtype

t = 4.1537, df = 42382, p-value = 3.278e-05

alternative hypothesis: true difference in means is not equal to 0

95 percent confidence interval:

0.1014097 0.2826281

sample estimates:

mean in group Ophthalmology mean in group Optometry

59.95618 59.76416

[1] 0.03949595

[1] "ayl_white_perc" "2012"

provtype value

1 Ophthalmology 82.00045

2 Optometry 87.77725

provtype value

1 Ophthalmology 22.87214

2 Optometry 23.49651

Welch Two Sample t-test

data: sdat$value by sdat$provtype

t = -20.084, df = 24486, p-value < 2.2e-16

alternative hypothesis: true difference in means is not equal to 0

95 percent confidence interval:

-6.340574 -5.213028

sample estimates:

mean in group Ophthalmology mean in group Optometry

82.00045 87.77725

[1] 0.2491458

[1] "ayl_white_perc" "2013"

provtype value

1 Ophthalmology 81.63239

2 Optometry 87.65367

provtype value

1 Ophthalmology 22.76043

2 Optometry 23.50833

Welch Two Sample t-test

data: sdat$value by sdat$provtype

t = -21.334, df = 25683, p-value < 2.2e-16

alternative hypothesis: true difference in means is not equal to 0

95 percent confidence interval:

-6.574478 -5.468064

sample estimates:

mean in group Ophthalmology mean in group Optometry

81.63239 87.65367

[1] 0.2602397

[1] "ayl_white_perc" "2014"

provtype value

1 Ophthalmology 81.19824

2 Optometry 87.77370

provtype value

1 Ophthalmology 22.48079

2 Optometry 23.30380

Welch Two Sample t-test

data: sdat$value by sdat$provtype

t = -23.964, df = 26911, p-value < 2.2e-16

alternative hypothesis: true difference in means is not equal to 0

95 percent confidence interval:

-7.113273 -6.037639

sample estimates:

mean in group Ophthalmology mean in group Optometry

81.19824 87.77370

[1] 0.287188

[1] "ayl_white_perc" "2015"

provtype value

1 Ophthalmology 80.75484

2 Optometry 88.17005

provtype value

1 Ophthalmology 22.49940

2 Optometry 22.29861

Welch Two Sample t-test

data: sdat$value by sdat$provtype

t = -28.1, df = 28506, p-value < 2.2e-16

alternative hypothesis: true difference in means is not equal to 0

95 percent confidence interval:

-7.932434 -6.897981

sample estimates:

mean in group Ophthalmology mean in group Optometry

80.75484 88.17005

[1] 0.3310475

[1] "ayl_white_perc" "2016"

provtype value

1 Ophthalmology 80.37414

2 Optometry 88.24675

provtype value

1 Ophthalmology 22.27187

2 Optometry 21.92422

Welch Two Sample t-test

data: sdat$value by sdat$provtype

t = -30.784, df = 29778, p-value < 2.2e-16

alternative hypothesis: true difference in means is not equal to 0

95 percent confidence interval:

-8.373864 -7.371350

sample estimates:

mean in group Ophthalmology mean in group Optometry

80.37414 88.24675

[1] 0.3562471

[1] "ayl_white_perc" "2017"

provtype value

1 Ophthalmology 80.08798

2 Optometry 88.34674

provtype value

1 Ophthalmology 22.35496

2 Optometry 21.45891

Welch Two Sample t-test

data: sdat$value by sdat$provtype

t = -32.904, df = 30469, p-value < 2.2e-16

alternative hypothesis: true difference in means is not equal to 0

95 percent confidence interval:

-8.750709 -7.766794

sample estimates:

mean in group Ophthalmology mean in group Optometry

80.08798 88.34674

[1] 0.3769138

[1] "ayl_black_perc" "2012"

provtype value

1 Ophthalmology 8.305460

2 Optometry 5.348304

provtype value

1 Ophthalmology 14.47909

2 Optometry 13.52726

Welch Two Sample t-test

data: sdat$value by sdat$provtype

t = 17.119, df = 25564, p-value < 2.2e-16

alternative hypothesis: true difference in means is not equal to 0

95 percent confidence interval:

2.618575 3.295735

sample estimates:

mean in group Ophthalmology mean in group Optometry

8.305460 5.348304

[1] 0.2110556

[1] "ayl_black_perc" "2013"

provtype value

1 Ophthalmology 8.351393

2 Optometry 5.401910

provtype value

1 Ophthalmology 14.40372

2 Optometry 13.55100

Welch Two Sample t-test

data: sdat$value by sdat$provtype

t = 17.379, df = 26634, p-value < 2.2e-16

alternative hypothesis: true difference in means is not equal to 0

95 percent confidence interval:

2.616827 3.282139

sample estimates:

mean in group Ophthalmology mean in group Optometry

8.351393 5.401910

[1] 0.2109204

[1] "ayl_black_perc" "2014"

provtype value

1 Ophthalmology 8.468819

2 Optometry 5.310841

provtype value

1 Ophthalmology 14.17379

2 Optometry 13.49241

Welch Two Sample t-test

data: sdat$value by sdat$provtype

t = 19.117, df = 27691, p-value < 2.2e-16

alternative hypothesis: true difference in means is not equal to 0

95 percent confidence interval:

2.834187 3.481770

sample estimates:

mean in group Ophthalmology mean in group Optometry

8.468819 5.310841

[1] 0.2282222

[1] "ayl_black_perc" "2015"

provtype value

1 Ophthalmology 8.552544

2 Optometry 5.250930

provtype value

1 Ophthalmology 14.36043

2 Optometry 13.12035

Welch Two Sample t-test

data: sdat$value by sdat$provtype

t = 20.429, df = 28878, p-value < 2.2e-16

alternative hypothesis: true difference in means is not equal to 0

95 percent confidence interval:

2.984845 3.618383

sample estimates:

mean in group Ophthalmology mean in group Optometry

8.552544 5.250930

[1] 0.2400411

[1] "ayl_black_perc" "2016"

provtype value

1 Ophthalmology 8.567646

2 Optometry 5.385905

provtype value

1 Ophthalmology 14.18467

2 Optometry 13.35492

Welch Two Sample t-test

data: sdat$value by sdat$provtype

t = 19.975, df = 29884, p-value < 2.2e-16

alternative hypothesis: true difference in means is not equal to 0

95 percent confidence interval:

2.869535 3.493947

sample estimates:

mean in group Ophthalmology mean in group Optometry

8.567646 5.385905

[1] 0.2309619

[1] "ayl_black_perc" "2017"

provtype value

1 Ophthalmology 8.485628

2 Optometry 5.226347

provtype value

1 Ophthalmology 14.01903

2 Optometry 12.78265

Welch Two Sample t-test

data: sdat$value by sdat$provtype

t = 21.222, df = 30402, p-value < 2.2e-16

alternative hypothesis: true difference in means is not equal to 0

95 percent confidence interval:

2.958260 3.560303

sample estimates:

mean in group Ophthalmology mean in group Optometry

8.485628 5.226347

[1] 0.2429563

[1] "ayl_hispanic_perc" "2012"

provtype value

1 Ophthalmology 6.226450

2 Optometry 4.140939

provtype value

1 Ophthalmology 14.35912

2 Optometry 14.05360

Welch Two Sample t-test

data: sdat$value by sdat$provtype

t = 11.871, df = 25075, p-value < 2.2e-16

alternative hypothesis: true difference in means is not equal to 0

95 percent confidence interval:

1.741153 2.429869

sample estimates:

mean in group Ophthalmology mean in group Optometry

6.226450 4.140939

[1] 0.1467927

[1] "ayl_hispanic_perc" "2013"

provtype value

1 Ophthalmology 6.265660

2 Optometry 4.136034

provtype value

1 Ophthalmology 14.12718

2 Optometry 13.78646

Welch Two Sample t-test

data: sdat$value by sdat$provtype

t = 12.546, df = 26303, p-value < 2.2e-16

alternative hypothesis: true difference in means is not equal to 0

95 percent confidence interval:

1.796926 2.462327

sample estimates:

mean in group Ophthalmology mean in group Optometry

6.265660 4.136034

[1] 0.1525754

[1] "ayl_hispanic_perc" "2014"

provtype value

1 Ophthalmology 6.314673

2 Optometry 3.908032

provtype value

1 Ophthalmology 14.06687

2 Optometry 13.08293

Welch Two Sample t-test

data: sdat$value by sdat$provtype

t = 14.856, df = 27843, p-value < 2.2e-16

alternative hypothesis: true difference in means is not equal to 0

95 percent confidence interval:

2.089113 2.724169

sample estimates:

mean in group Ophthalmology mean in group Optometry

6.314673 3.908032

[1] 0.1771698

[1] "ayl_hispanic_perc" "2015"

provtype value

1 Ophthalmology 6.342677

2 Optometry 3.812741

provtype value

1 Ophthalmology 13.96573

2 Optometry 12.58442

Welch Two Sample t-test

data: sdat$value by sdat$provtype

t = 16.205, df = 28904, p-value < 2.2e-16

alternative hypothesis: true difference in means is not equal to 0

95 percent confidence interval:

2.223929 2.835944

sample estimates:

mean in group Ophthalmology mean in group Optometry

6.342677 3.812741

[1] 0.1903206

[1] "ayl_hispanic_perc" "2016"

provtype value

1 Ophthalmology 6.345572

2 Optometry 3.602881

provtype value

1 Ophthalmology 13.64985

2 Optometry 12.01171

Welch Two Sample t-test

data: sdat$value by sdat$provtype

t = 18.474, df = 29819, p-value < 2.2e-16

alternative hypothesis: true difference in means is not equal to 0

95 percent confidence interval:

2.451697 3.033685

sample estimates:

mean in group Ophthalmology mean in group Optometry

6.345572 3.602881

[1] 0.2133246

[1] "ayl_hispanic_perc" "2017"

provtype value

1 Ophthalmology 6.340075

2 Optometry 3.473649

provtype value

1 Ophthalmology 13.78241

2 Optometry 11.44674

Welch Two Sample t-test

data: sdat$value by sdat$provtype

t = 19.784, df = 29890, p-value < 2.2e-16

alternative hypothesis: true difference in means is not equal to 0

95 percent confidence interval:

2.582450 3.150402

sample estimates:

mean in group Ophthalmology mean in group Optometry

6.340075 3.473649

[1] 0.2262637

[1] "ayl_api_perc" "2012"

provtype value

1 Ophthalmology 2.876868

2 Optometry 1.567884

provtype value

1 Ophthalmology 10.445673

2 Optometry 9.634687

Welch Two Sample t-test

data: sdat$value by sdat$provtype

t = 10.575, df = 25684, p-value < 2.2e-16

alternative hypothesis: true difference in means is not equal to 0

95 percent confidence interval:

1.066370 1.551598

sample estimates:

mean in group Ophthalmology mean in group Optometry

2.876868 1.567884

[1] 0.1302683

[1] "ayl_api_perc" "2013"

provtype value

1 Ophthalmology 3.006702

2 Optometry 1.523068

provtype value

1 Ophthalmology 10.558032

2 Optometry 9.305006

Welch Two Sample t-test

data: sdat$value by sdat$provtype

t = 12.328, df = 27067, p-value < 2.2e-16

alternative hypothesis: true difference in means is not equal to 0

95 percent confidence interval:

1.247747 1.719520

sample estimates:

mean in group Ophthalmology mean in group Optometry

3.006702 1.523068

[1] 0.14909

[1] "ayl_api_perc" "2014"

provtype value

1 Ophthalmology 3.073208

2 Optometry 1.560692

provtype value

1 Ophthalmology 10.455906

2 Optometry 9.403306

Welch Two Sample t-test

data: sdat$value by sdat$provtype

t = 12.774, df = 28013, p-value < 2.2e-16

alternative hypothesis: true difference in means is not equal to 0

95 percent confidence interval:

1.280432 1.744601

sample estimates:

mean in group Ophthalmology mean in group Optometry

3.073208 1.560692

[1] 0.1521104

[1] "ayl_api_perc" "2015"

provtype value

1 Ophthalmology 3.189834

2 Optometry 1.424799

provtype value

1 Ophthalmology 10.56288

2 Optometry 8.53659

Welch Two Sample t-test

data: sdat$value by sdat$provtype

t = 15.704, df = 28728, p-value < 2.2e-16

alternative hypothesis: true difference in means is not equal to 0

95 percent confidence interval:

1.544732 1.985340

sample estimates:

mean in group Ophthalmology mean in group Optometry

3.189834 1.424799

[1] 0.1837942

[1] "ayl_api_perc" "2016"

provtype value

1 Ophthalmology 3.315474

2 Optometry 1.335755

provtype value

1 Ophthalmology 10.645477

2 Optometry 8.145683

Welch Two Sample t-test

data: sdat$value by sdat$provtype

t = 18.136, df = 28894, p-value < 2.2e-16

alternative hypothesis: true difference in means is not equal to 0

95 percent confidence interval:

1.765757 2.193681

sample estimates:

mean in group Ophthalmology mean in group Optometry

3.315474 1.335755

[1] 0.2088674

[1] "ayl_api_perc" "2017"

provtype value

1 Ophthalmology 3.406595

2 Optometry 1.401308

provtype value

1 Ophthalmology 10.813702

2 Optometry 8.253904

Welch Two Sample t-test

data: sdat$value by sdat$provtype

t = 18.245, df = 29061, p-value < 2.2e-16

alternative hypothesis: true difference in means is not equal to 0

95 percent confidence interval:

1.789856 2.220717

sample estimates:

mean in group Ophthalmology mean in group Optometry

3.406595 1.401308

[1] 0.2084643

[1] "ayl_natind_perc" "2012"

provtype value

1 Ophthalmology 0.1721785

2 Optometry 1.0461312

provtype value

1 Ophthalmology 2.332737

2 Optometry 9.579169

Welch Two Sample t-test

data: sdat$value by sdat$provtype

t = -9.5849, df = 12597, p-value < 2.2e-16

alternative hypothesis: true difference in means is not equal to 0

95 percent confidence interval:

-1.052679 -0.695226

sample estimates:

mean in group Ophthalmology mean in group Optometry

0.1721785 1.0461312

[1] 0.1253617

[1] "ayl_natind_perc" "2013"

provtype value

1 Ophthalmology 0.1658836

2 Optometry 1.1252778

provtype value

1 Ophthalmology 2.183435

2 Optometry 10.011086

Welch Two Sample t-test

data: sdat$value by sdat$provtype

t = -10.374, df = 13098, p-value < 2.2e-16

alternative hypothesis: true difference in means is not equal to 0

95 percent confidence interval:

-1.1406724 -0.7781162

sample estimates:

mean in group Ophthalmology mean in group Optometry

0.1658836 1.1252778

[1] 0.1324158

[1] "ayl_natind_perc" "2014"

provtype value

1 Ophthalmology 0.1938578

2 Optometry 1.2303042

provtype value

1 Ophthalmology 2.55225

2 Optometry 10.48932

Welch Two Sample t-test

data: sdat$value by sdat$provtype

t = -10.921, df = 14089, p-value < 2.2e-16

alternative hypothesis: true difference in means is not equal to 0

95 percent confidence interval:

-1.2224708 -0.8504219

sample estimates:

mean in group Ophthalmology mean in group Optometry

0.1938578 1.2303042

[1] 0.1357765

[1] "ayl_natind_perc" "2015"

provtype value

1 Ophthalmology 0.1827974

2 Optometry 1.0592155

provtype value

1 Ophthalmology 2.369129

2 Optometry 9.633358

Welch Two Sample t-test

data: sdat$value by sdat$provtype

t = -10.31, df = 14962, p-value < 2.2e-16

alternative hypothesis: true difference in means is not equal to 0

95 percent confidence interval:

-1.0430395 -0.7097967

sample estimates:

mean in group Ophthalmology mean in group Optometry

0.1827974 1.0592155

[1] 0.1249387

[1] "ayl_natind_perc" "2016"

provtype value

1 Ophthalmology 0.1878892

2 Optometry 1.0456610

provtype value

1 Ophthalmology 2.396575

2 Optometry 9.583584

Welch Two Sample t-test

data: sdat$value by sdat$provtype

t = -10.43, df = 16024, p-value < 2.2e-16

alternative hypothesis: true difference in means is not equal to 0

95 percent confidence interval:

-1.018972 -0.696571

sample estimates:

mean in group Ophthalmology mean in group Optometry

0.1878892 1.0456610

[1] 0.1227968

[1] "ayl_natind_perc" "2017"

provtype value

1 Ophthalmology 0.1714321

2 Optometry 1.0393520

provtype value

1 Ophthalmology 1.920832

2 Optometry 9.503280

Welch Two Sample t-test

data: sdat$value by sdat$provtype

t = -10.934, df = 16055, p-value < 2.2e-16

alternative hypothesis: true difference in means is not equal to 0

95 percent confidence interval:

-1.0235103 -0.7123297

sample estimates:

mean in group Ophthalmology mean in group Optometry

0.1714321 1.0393520

[1] 0.1265978

[1] "ayl_other_perc" "2012"

provtype value

1 Ophthalmology 0.4185446

2 Optometry 0.1195009

provtype value

1 Ophthalmology 1.347446

2 Optometry 1.122733

Welch Two Sample t-test

data: sdat$value by sdat$provtype

t = 19.704, df = 26366, p-value < 2.2e-16

alternative hypothesis: true difference in means is not equal to 0

95 percent confidence interval:

0.2692968 0.3287905

sample estimates:

mean in group Ophthalmology mean in group Optometry

0.4185446 0.1195009

[1] 0.2411274

[1] "ayl_other_perc" "2013"

provtype value

1 Ophthalmology 0.5779877

2 Optometry 0.1600559

provtype value

1 Ophthalmology 1.491791

2 Optometry 1.204438

Welch Two Sample t-test

data: sdat$value by sdat$provtype

t = 25.61, df = 27296, p-value < 2.2e-16

alternative hypothesis: true difference in means is not equal to 0

95 percent confidence interval:

0.3859458 0.4499178

sample estimates:

mean in group Ophthalmology mean in group Optometry

0.5779877 0.1600559

[1] 0.3082664

[1] "ayl_other_perc" "2014"

provtype value

1 Ophthalmology 0.7511891

2 Optometry 0.2164165

provtype value

1 Ophthalmology 1.622296

2 Optometry 1.325242

Welch Two Sample t-test

data: sdat$value by sdat$provtype

t = 30.448, df = 28152, p-value < 2.2e-16

alternative hypothesis: true difference in means is not equal to 0

95 percent confidence interval:

0.5003475 0.5691976

sample estimates:

mean in group Ophthalmology mean in group Optometry

0.7511891 0.2164165

[1] 0.3610317

[1] "ayl_other_perc" "2015"

provtype value

1 Ophthalmology 0.9772864

2 Optometry 0.2822691

provtype value

1 Ophthalmology 1.796933

2 Optometry 1.429313

Welch Two Sample t-test

data: sdat$value by sdat$provtype

t = 36.594, df = 28648, p-value < 2.2e-16

alternative hypothesis: true difference in means is not equal to 0

95 percent confidence interval:

0.6577907 0.7322438

sample estimates:

mean in group Ophthalmology mean in group Optometry

0.9772864 0.2822691

[1] 0.4280818

[1] "ayl_other_perc" "2016"

provtype value

1 Ophthalmology 1.2092453

2 Optometry 0.3830804

provtype value

1 Ophthalmology 1.938748

2 Optometry 1.600094

Welch Two Sample t-test

data: sdat$value by sdat$provtype

t = 40.3, df = 29517, p-value < 2.2e-16

alternative hypothesis: true difference in means is not equal to 0

95 percent confidence interval:

0.7859834 0.8663465

sample estimates:

mean in group Ophthalmology mean in group Optometry

1.2092453 0.3830804

[1] 0.4647892

[1] "ayl_other_perc" "2017"

provtype value

1 Ophthalmology 1.5082637

2 Optometry 0.5126086

provtype value

1 Ophthalmology 2.173593

2 Optometry 1.829317

Welch Two Sample t-test

data: sdat$value by sdat$provtype

t = 43.332, df = 29991, p-value < 2.2e-16

alternative hypothesis: true difference in means is not equal to 0

95 percent confidence interval:

0.9506185 1.0406916

sample estimates:

mean in group Ophthalmology mean in group Optometry

1.5082637 0.5126086

[1] 0.4956358

[1] "TOTAL_UNIQUE_BENES" "2012"

provtype value

1 Ophthalmology 457.2020

2 Optometry 197.8194

provtype value

1 Ophthalmology 286.4507

2 Optometry 188.5795

Welch Two Sample t-test

data: sdat$value by sdat$provtype

t = 92.093, df = 17664, p-value < 2.2e-16

alternative hypothesis: true difference in means is not equal to 0

95 percent confidence interval:

253.8620 264.9033

sample estimates:

mean in group Ophthalmology mean in group Optometry

457.2020 197.8194

[1] NA

[1] "TOTAL_UNIQUE_BENES" "2013"

provtype value

1 Ophthalmology 456.8024

2 Optometry 198.5078

provtype value

1 Ophthalmology 287.6883

2 Optometry 188.2625

Welch Two Sample t-test

data: sdat$value by sdat$provtype

t = 92.07, df = 17735, p-value < 2.2e-16

alternative hypothesis: true difference in means is not equal to 0

95 percent confidence interval:

252.7957 263.7935

sample estimates:

mean in group Ophthalmology mean in group Optometry

456.8024 198.5078

[1] NA

[1] "TOTAL_UNIQUE_BENES" "2014"

provtype value

1 Ophthalmology 733.7506

2 Optometry 223.7254

provtype value

1 Ophthalmology 563.5749

2 Optometry 268.4844

Welch Two Sample t-test

data: sdat$value by sdat$provtype

t = 112.61, df = 22710, p-value < 2.2e-16

alternative hypothesis: true difference in means is not equal to 0

95 percent confidence interval:

501.1480 518.9023

sample estimates:

mean in group Ophthalmology mean in group Optometry

733.7506 223.7254

[1] 1.155423

[1] "TOTAL_UNIQUE_BENES" "2015"

provtype value

1 Ophthalmology 731.9936

2 Optometry 227.0262

provtype value

1 Ophthalmology 562.4094

2 Optometry 267.7812

Welch Two Sample t-test

data: sdat$value by sdat$provtype

t = 111.91, df = 22695, p-value < 2.2e-16

alternative hypothesis: true difference in means is not equal to 0

95 percent confidence interval:

496.1233 513.8115

sample estimates:

mean in group Ophthalmology mean in group Optometry

731.9936 227.0262

[1] 1.146453

[1] "TOTAL_UNIQUE_BENES" "2016"

provtype value

1 Ophthalmology 734.0477

2 Optometry 232.0802

provtype value

1 Ophthalmology 566.0616

2 Optometry 273.4589

Welch Two Sample t-test

data: sdat$value by sdat$provtype

t = 110.67, df = 22917, p-value < 2.2e-16

alternative hypothesis: true difference in means is not equal to 0

95 percent confidence interval:

493.0773 510.8577

sample estimates:

mean in group Ophthalmology mean in group Optometry

734.0477 232.0802

[1] 1.129221

[1] "TOTAL_UNIQUE_BENES" "2017"

provtype value

1 Ophthalmology 722.3003

2 Optometry 234.2702

provtype value

1 Ophthalmology 558.6933

2 Optometry 275.5095

Welch Two Sample t-test

data: sdat$value by sdat$provtype

t = 108.94, df = 23083, p-value < 2.2e-16

alternative hypothesis: true difference in means is not equal to 0

95 percent confidence interval:

479.2497 496.8105

sample estimates:

mean in group Ophthalmology mean in group Optometry

722.3003 234.2702

[1] 1.107953

[1] "BENEFICIARY_AVERAGE_AGE" "TOTAL"

provtype value

1 Ophthalmology 74.55753

2 Optometry 72.30219

provtype value

1 Ophthalmology 3.095141

2 Optometry 3.469009

Welch Two Sample t-test

data: sdat$value by sdat$provtype

t = 177.92, df = 243515, p-value < 2.2e-16

alternative hypothesis: true difference in means is not equal to 0

95 percent confidence interval:

2.230500 2.280189

sample estimates:

mean in group Ophthalmology mean in group Optometry

74.55753 72.30219

[1] 0.6860585

[1] "ayl_female_perc" "TOTAL"

provtype value

1 Ophthalmology 60.22944

2 Optometry 59.87207

provtype value

1 Ophthalmology 4.178271

2 Optometry 5.589779

Welch Two Sample t-test

data: sdat$value by sdat$provtype

t = 18.488, df = 252962, p-value < 2.2e-16

alternative hypothesis: true difference in means is not equal to 0

95 percent confidence interval:

0.3194861 0.3952584

sample estimates:

mean in group Ophthalmology mean in group Optometry

60.22944 59.87207

[1] 0.07241948

[1] "ayl_white_perc" "TOTAL"

provtype value

1 Ophthalmology 80.99899

2 Optometry 88.01668

provtype value

1 Ophthalmology 22.54753

2 Optometry 22.61110

Welch Two Sample t-test

data: sdat$value by sdat$provtype

t = -64.133, df = 167463, p-value < 2.2e-16

alternative hypothesis: true difference in means is not equal to 0

95 percent confidence interval:

-7.232155 -6.803219

sample estimates:

mean in group Ophthalmology mean in group Optometry

80.99899 88.01668

[1] 0.3108013

[1] "ayl_black_perc" "TOTAL"

provtype value

1 Ophthalmology 8.456426

2 Optometry 5.317775

provtype value

1 Ophthalmology 14.26906

2 Optometry 13.28757

Welch Two Sample t-test

data: sdat$value by sdat$provtype

t = 47.105, df = 170279, p-value < 2.2e-16

alternative hypothesis: true difference in means is not equal to 0

95 percent confidence interval:

3.008054 3.269247

sample estimates:

mean in group Ophthalmology mean in group Optometry

8.456426 5.317775

[1] 0.2276521

[1] "ayl_hispanic_perc" "TOTAL"

provtype value

1 Ophthalmology 6.306438

2 Optometry 3.823809

provtype value

1 Ophthalmology 13.99050

2 Optometry 12.78125

Welch Two Sample t-test

data: sdat$value by sdat$provtype

t = 38.364, df = 170718, p-value < 2.2e-16

alternative hypothesis: true difference in means is not equal to 0

95 percent confidence interval:

2.355793 2.609465

sample estimates:

mean in group Ophthalmology mean in group Optometry

6.306438 3.823809

[1] 0.1852775

[1] "ayl_api_perc" "TOTAL"

provtype value

1 Ophthalmology 3.147162

2 Optometry 1.462122

provtype value

1 Ophthalmology 10.58355

2 Optometry 8.85006

Welch Two Sample t-test

data: sdat$value by sdat$provtype

t = 35.882, df = 171130, p-value < 2.2e-16

alternative hypothesis: true difference in means is not equal to 0

95 percent confidence interval:

1.592998 1.777083

sample estimates:

mean in group Ophthalmology mean in group Optometry

3.147162 1.462122

[1] 0.1727293

[1] "ayl_natind_perc" "TOTAL"

provtype value

1 Ophthalmology 0.1790644

2 Optometry 1.0889158

provtype value

1 Ophthalmology 2.300510

2 Optometry 9.794408

Welch Two Sample t-test

data: sdat$value by sdat$provtype

t = -25.56, df = 86864, p-value < 2.2e-16

alternative hypothesis: true difference in means is not equal to 0

95 percent confidence interval:

-0.9796199 -0.8400829

sample estimates:

mean in group Ophthalmology mean in group Optometry

0.1790644 1.0889158

[1] 0.1278929

[1] "ayl_other_perc" "TOTAL"

provtype value

1 Ophthalmology 0.9118971

2 Optometry 0.2907046

provtype value

1 Ophthalmology 1.792949

2 Optometry 1.465884

Welch Two Sample t-test

data: sdat$value by sdat$provtype

t = 78.864, df = 170813, p-value < 2.2e-16

alternative hypothesis: true difference in means is not equal to 0

95 percent confidence interval:

0.6057543 0.6366308

sample estimates:

mean in group Ophthalmology mean in group Optometry

0.9118971 0.2907046

[1] 0.3793306

[1] "TOTAL_UNIQUE_BENES" "TOTAL"

provtype value

1 Ophthalmology 659.1879

2 Optometry 219.4738

provtype value

1 Ophthalmology 519.5856

2 Optometry 248.5770

Welch Two Sample t-test

data: sdat$value by sdat$provtype

t = 246.77, df = 121250, p-value < 2.2e-16

alternative hypothesis: true difference in means is not equal to 0

95 percent confidence interval:

436.2216 443.2066

sample estimates:

mean in group Ophthalmology mean in group Optometry

659.1879 219.4738

[1] NA

[1] "BENEFICIARY_AVERAGE_RISK_SCORE" "2012"

provtype value

1 Ophthalmology 1.246815

2 Optometry 1.078594

provtype value

1 Ophthalmology 0.2836778

2 Optometry 0.2926742

Welch Two Sample t-test

data: sdat$value by sdat$provtype

t = 60.301, df = 37933, p-value < 2.2e-16

alternative hypothesis: true difference in means is not equal to 0

95 percent confidence interval:

0.1627539 0.1736897

sample estimates:

mean in group Ophthalmology mean in group Optometry

1.246815 1.078594

[1] 0.5836756

[1] "BENEFICIARY_AVERAGE_RISK_SCORE" "2013"

provtype value

1 Ophthalmology 1.219316

2 Optometry 1.057567

provtype value

1 Ophthalmology 0.2679149

2 Optometry 0.2932556

Welch Two Sample t-test

data: sdat$value by sdat$provtype

t = 60.396, df = 39762, p-value < 2.2e-16

alternative hypothesis: true difference in means is not equal to 0

95 percent confidence interval:

0.1564995 0.1669979

sample estimates:

mean in group Ophthalmology mean in group Optometry

1.219316 1.057567

[1] 0.5758822

[1] "BENEFICIARY_AVERAGE_RISK_SCORE" "2014"

provtype value

1 Ophthalmology 1.202842

2 Optometry 1.032815

provtype value

1 Ophthalmology 0.2723797

2 Optometry 0.2939570

Welch Two Sample t-test

data: sdat$value by sdat$provtype

t = 63.211, df = 39591, p-value < 2.2e-16

alternative hypothesis: true difference in means is not equal to 0

95 percent confidence interval:

0.1647553 0.1752996

sample estimates:

mean in group Ophthalmology mean in group Optometry

1.202842 1.032815

[1] 0.6000112

[1] "BENEFICIARY_AVERAGE_RISK_SCORE" "2015"

provtype value

1 Ophthalmology 1.264108

2 Optometry 1.086654

provtype value

1 Ophthalmology 0.2853817

2 Optometry 0.3082973

Welch Two Sample t-test

data: sdat$value by sdat$provtype

t = 63.143, df = 39645, p-value < 2.2e-16

alternative hypothesis: true difference in means is not equal to 0

95 percent confidence interval:

0.1719461 0.1829629

sample estimates:

mean in group Ophthalmology mean in group Optometry

1.264108 1.086654

[1] 0.5973682

[1] "BENEFICIARY_AVERAGE_RISK_SCORE" "2016"

provtype value

1 Ophthalmology 1.276946

2 Optometry 1.090572

provtype value

1 Ophthalmology 0.2980352

2 Optometry 0.3161699

Welch Two Sample t-test

data: sdat$value by sdat$provtype

t = 64.253, df = 39282, p-value < 2.2e-16

alternative hypothesis: true difference in means is not equal to 0

95 percent confidence interval:

0.1806887 0.1920594

sample estimates:

mean in group Ophthalmology mean in group Optometry

1.276946 1.090572

[1] 0.6066146

[1] "BENEFICIARY_AVERAGE_RISK_SCORE" "2017"

provtype value

1 Ophthalmology 1.290189

2 Optometry 1.093851

provtype value

1 Ophthalmology 0.3216220

2 Optometry 0.3102745

Welch Two Sample t-test

data: sdat$value by sdat$provtype

t = 65.31, df = 36421, p-value < 2.2e-16

alternative hypothesis: true difference in means is not equal to 0

95 percent confidence interval:

0.1904454 0.2022299

sample estimates:

mean in group Ophthalmology mean in group Optometry

1.290189 1.093851

[1] 0.6213233

[1] "BENEFICIARY_AVERAGE_RISK_SCORE" "TOTAL"

provtype value

1 Ophthalmology 1.250171

2 Optometry 1.073616

provtype value

1 Ophthalmology 0.2904552

2 Optometry 0.3035992

Welch Two Sample t-test

data: sdat$value by sdat$provtype

t = 152.84, df = 231913, p-value < 2.2e-16

alternative hypothesis: true difference in means is not equal to 0

95 percent confidence interval:

0.1742907 0.1788189

sample estimates:

mean in group Ophthalmology mean in group Optometry

1.250171 1.073616

[1] 0.5942607
